# Clinical and economic implications of epilepsy management across treatment lines in Spain: a real-life database analysis

**DOI:** 10.1007/s00415-023-11958-x

**Published:** 2023-08-25

**Authors:** Rafael Toledano, Vicente Villanueva, Manuel Toledo, Joel Sabaniego, Paloma Pérez-Domper

**Affiliations:** 1 Epilepsy Unit, Neurology Service, Hospital Universitario Ramón y Cajal and Hospital Ruber Internacional, Madrid, Spain; 2 Refractory Epilepsy Unit, Neurology Service, Hospital Universitario y Politécnico La Fe, Member of ERN EpiCARE, Valencia, Spain; 3 Neurology Service, Hospital Universitari Vall d’ Hebron, Barcelona, Spain; 4 Angelini Pharma, Barcelona, Spain

**Keywords:** Drug-resistant epilepsy, Healthcare resources, Treatment line, Antiseizure medications, Real-life data

## Abstract

**Background:**

Epilepsy is a chronic brain disease characterized by recurrent seizures. We investigated real-world management of epilepsy across treatment lines in Spain, including healthcare resource use (HRU) and associated costs.

**Methods:**

This was a retrospective study of real-life data from epilepsy patients prescribed antiseizure medication (ASM) between January 2016 and December 2021. Patients were grouped according to their line of treatment (1st, 2nd, 3rd and 4th +) during the recruitment period. Demographic and clinical characteristics, comorbidities and concomitant medications were analyzed during the baseline period (6 months before starting treatment line); antiepileptic treatments, concomitant medications, HRU and associated costs were analyzed during follow-up.

**Results:**

The study included 5006 patients. Treatment duration decreased as treatment lines progressed (mean ± SD progression time: 523.2 ± 279.1 days from 1st to 2nd line, 351.6 ± 194.4 days from 2nd to 3rd line; 272.7 ± 139.3 days from 3rd to 4th + line). Significant HRU differences were found with subsequent treatment lines, including an increase in hospital admissions and patients on sick leave. Mean (95% CI) adjusted total costs per patient were €2974/year (2773–3175) in the 1st line and €5735/year (5043–6428) in the 4th + line. There was an increase in adjusted direct and total costs with subsequent treatment lines; the mean difference in total costs between cohorts was €2761 (*p* < 0.001). The highest direct costs were associated with epilepsy medication, days at the hospital and specialist visits.

**Conclusion:**

Our data revealed a progressive increase in the use of resources and associated costs across subsequent epilepsy treatment lines.

**Supplementary Information:**

The online version contains supplementary material available at 10.1007/s00415-023-11958-x.

## Introduction

Epilepsy is a chronic brain disease characterized by recurrent seizures and is one of the most common neurologic diseases, with approximately 50 million people affected worldwide [1, 2]. The annual incidence rate of epilepsy has been estimated at 50.4 to 81.7 cases per 100,000 individuals [1, 3], and according to the Global Burden of Disease Study, epilepsy constitutes a significant cause of disability and mortality [4]. In Spain, the mortality rate of patients with epilepsy is estimated to be two to three times higher than that of the general population [5]. In addition, patients with epilepsy present with many comorbidities, such as depression, anxiety, dementia, and migraine [6].

The ultimate epilepsy treatment goals are to achieve seizure freedom without clinically significant adverse effects and to improve quality of life [5, 7, 8]. Epilepsy guidelines recommend a first-line treatment based on monotherapy, followed by a second monotherapy or a first adjuvant treatment if seizures persist [5]. Nonetheless, existing guidelines do not contain therapeutic algorithms recommending the use of specific pharmacological alternatives for each treatment line [5, 8, 10].

Drug-resistant epilepsy (DRE) is defined by the International League Against Epilepsy (ILAE) as the failure of two tolerated, appropriately chosen and used antiepileptic drug schedules (whether as monotherapies or in combination) to achieve sustained seizure freedom [6, 11]. In recent decades, the number of antiseizure medications (ASMs) has increased and many therapeutic alternatives are now available [5], but studies suggest that 30–40% of patients can still be defined as drug-resistant based on the ILAE definition [12–14]. Poor medication adherence and comorbidities are key predictors of lack of seizure control [15].

The costs associated with epilepsy management depend on disease duration and severity, response to treatment, and healthcare setting [16–18]. Consequently, patients with poor control and more severe forms of epilepsy, such as DRE, use more healthcare resources, resulting in substantial costs for healthcare systems [17–19]. In addition, epilepsy causes significant productivity losses derived from the higher frequency of unemployment and precariousness among patients [16, 20]. Despite the existing evidence regarding the clinical and economic implications of epilepsy, data on the management of patients across treatment lines is limited [21]. In Spain, real-life studies regarding epilepsy management, healthcare resource use, and associated costs for the Spanish National Health System (SNHS) and society are scarce. This 6-year, retrospective study aimed to analyze the clinical and economic consequences of the current management of adult patients with epilepsy across treatment lines in Spain using clinical practice data from a large administrative database collected between January 2016 and December 2021. To that end, we studied: the demographic characteristics and comorbidities of patients with epilepsy across treatment lines; the epilepsy treatments and concomitant medications used in clinical practice; and the use of healthcare resources and associated costs.

## Methods

### Study design

This was a retrospective, observational study to assess clinical and economic consequences of current epilepsy management across treatment lines in Spain. Data from adult patients with epilepsy who started an ASM between January 2016 and December 2021 (recruitment period) were obtained from electronic medical records (EMRs) collated within the BIG-PAC^®^ administrative database.

BIG-PAC^®^ is a dissociated and anonymous administrative database. It contains the integrated records of GP visits (primary care), emergency care, pharmacy dispensements/prescriptions (verified daily dose record, time interval, and duration of each treatment administered), hospital admissions, working days lost and disability data, and deaths data collected since 2012 from the computerized medical records of seven integrated public health areas of Spain covering 1.9 million patients. BIG-PAC^®^ is registered with the European Network of Centers for Pharmacoepidemiology and Pharmacovigilance with dependency of the European Medicines Agency (EMA) and has shown representativeness of the Spanish population [22, 23]. Before exporting to BIG-PAC^®^, primary data collected in EMRs were anonymized at the center of origin, in compliance with Organic Law 3/2018 of December 5 on the Protection of Personal Data and guarantee of digital rights [24].

The study was approved by the Ethics Committee of the Consorci Sanitari of Terrassa. Furthermore, it was developed following the ethical principles originating from the latest version of the Declaration of Helsinki accepted by local authorities and which are in line with Good Clinical Practice (GCP) and the requirements of current Spanish regulations. This study followed the requirements of the Reporting of studies Conducted using Observational Routinely-collected health Data (RECORD) [25].

### Inclusion and exclusion criteria

Inclusion criteria were: (a) 18 years old or older; (b) epilepsy diagnosis (defined according to the International Classification of Disease, 9th Revision, Clinical Modification [ICD-9-CM] code: 345 [26]); (c) being active patient in the database for a minimum of 12 months before starting the study; (d) inclusion in the chronic prescription program to obtain medical prescriptions (with a verified record of the daily dose, the time interval, and the duration of each treatment administered; ≥ 2 prescriptions during the follow-up period); having a regular follow up with ≥ 2 health records in the system computer. Exclusion criteria were: (a) transference to other centers; (b) relocation; (c) being permanently institutionalized; and (d) suffering a terminal disease and/or being treated with dialysis.

### Study cohorts

**Fig. 1:**
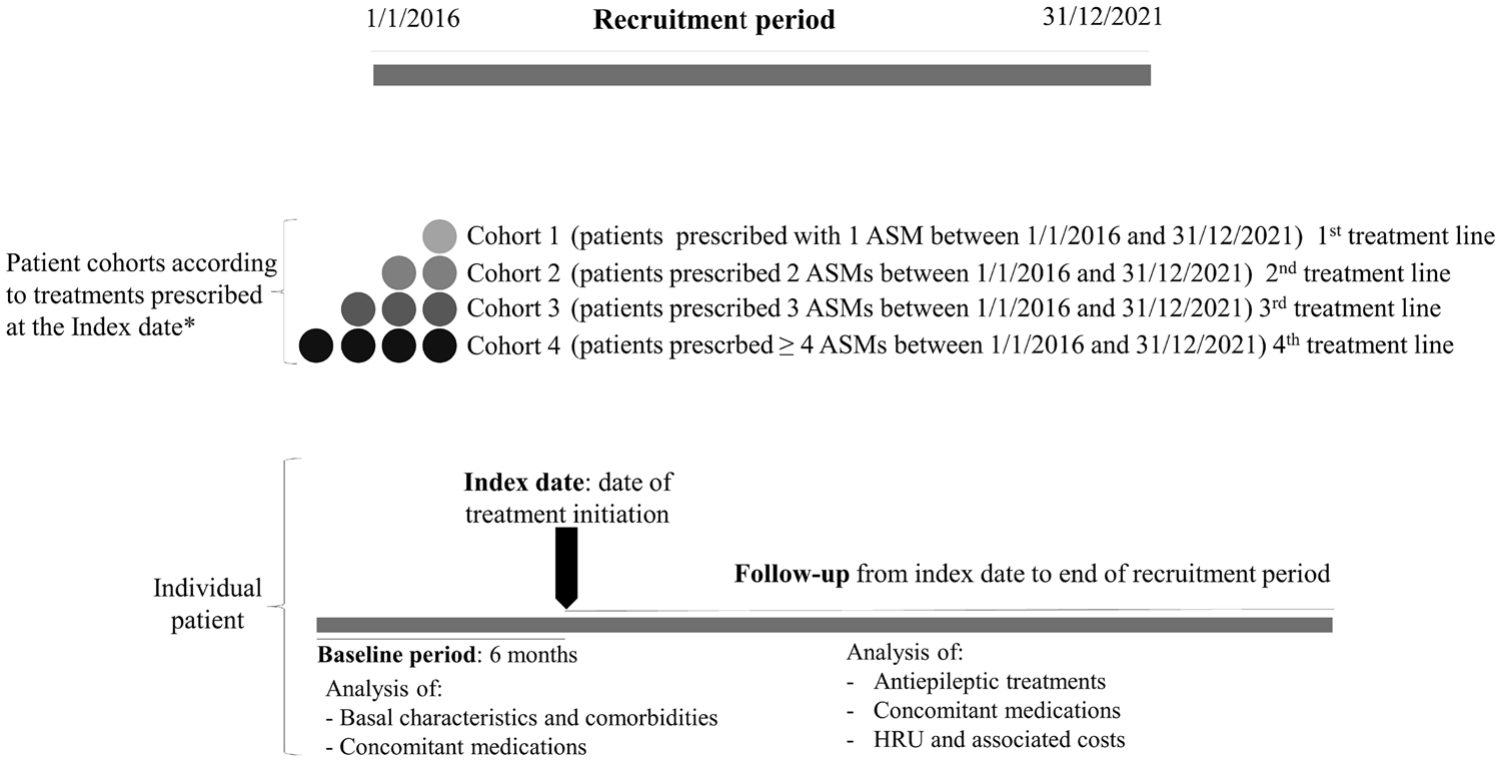
Study diagram. *Only treatments that lasted more than 3 months were considered to establish the sequence of treatment. Stratification by treatment was carried out in all patients recruited in the study. If, for instance, a patient was treated with 1 ASM, then with 2 ASMs, and finally with 3 ASMs during the recruitment period, the index date for that patient would be the date when 3 ASMs were prescribed and the cohort in which that patient was included for the analysis would be the cohort of patients treated with 3 ASMs. ASM, antiseizure medication; HRU, health resource use

The study population was divided into four groups according to the number of ASMs that they had been prescribed at the index date, as follows: cohort 1, patients on first-line treatment; cohort 2, patients on second-line treatment; cohort 3, patients on third-line treatment; and cohort 4, patients on fourth line (or above) treatment (Fig. 1). The index date was defined as the date of initiation of the last treatment line received by the patient within the recruitment period. Patients were followed up from the index date until December 2021. The EMRs of selected patients were also retrospectively reviewed to assess the comorbidities and concomitant medications of the patient during the baseline period (6 months before the index date).

### Study endpoints

Study endpoints included demographic characteristics, comorbidities, treatment information, and healthcare resource use and costs.

### Baseline parameters

Demographic variables (age and sex) were recorded at the index date. Comorbidities registered within six months before the index date were recorded using the ICD-9-CM coding system [26] (Table S1), including hypertension, diabetes, dyslipidemia, obesity, active smoking, alcohol ingestion, ischemic heart disease, cerebrovascular accident, heart failure, kidney failure, asthma, chronic obstructive pulmonary disease, depressive syndrome, and malignant neoplasms. Other comorbidities, such as anxiety, psychoses, and attention deficit disorder with hyperactivity, were also considered. In addition, the Charlson comorbidity index [27] was calculated to obtain a general comorbidity composite variable and a proxy to severity (Table S2).

### Treatment information

Treatment information was obtained from drug dispensing records. Physicians chose drugs for each specific patient at their discretion, according to clinical practice. Drugs were collected using the Anatomical Therapeutic Chemical (ATC) Classification System [28] as follows: antiepileptics (N03), psycholeptics (N05; including antipsychotics, anxiolytics, hypnotics and sedatives), and psychoanaleptics (N06; including antidepressants and stimulants). Treatment durations of less than three months were considered as withdrawn. Therefore, only antiepileptic treatments lasting more than three months were considered to establish a treatment sequence. The main ASMs considered are summarized in Table 3. Concomitant medications considered in the analysis are shown in Table 4.

### Healthcare resource use and costs

Healthcare resource use included visits to primary care, emergency services and specialists (including psychiatry, psychology and neurology), hospitalizations (annual rate and length of stay [days]), laboratory and diagnostic tests (conventional radiology, computed tomography, nuclear magnetic resonance, and electroencephalography), and medication. Hospitalizations also included admissions for surgical procedures, as described in Table S3. The healthcare resources consumed during the follow-up period were normalized per patient and year.

Healthcare costs (i.e., direct costs) were calculated considering the frequency of use during the follow-up and their unit cost for 2020 (based on hospital accounting) (Table S4). Medical prescriptions were quantified according to the retail price per pack at the time of prescription [29]. Non-healthcare costs (i.e., indirect costs) included those associated with productivity loss (i.e., absenteeism) and were measured as cost per day of sick leave due to temporary or permanent disability in the working population (< 65 years), considering the number of sick leave days/permanent disability days and the mean salary of the Spanish population, according to the National Institute of Statistics (Instituto Nacional de Estadística. n.d.) (Table S4).

### Statistical analysis

SQL scripts were used for BIG-PAC data extraction. The data were carefully reviewed through exploratory analysis and preparation of data for analysis by observing the frequency distributions and searching for possible recording or coding errors. Data validation was carried out to ensure the quality of the results. SQL and MS Access were used for data processing and statistical analysis, including data collection, retrieval, and preparation procedures. Qualitative variables were described using absolute and relative frequencies (N, %), and quantitative variables using the mean and standard deviation (SD), median and interquartile ranges (IQR: P25–P75; Q1–Q3), and confidence intervals of 95% (95% CI). Bivariate analyses were performed using analysis of variance (ANOVA), Chi-square tests, and Pearson linear correlation. Analysis of covariance (ANCOVA) was used to adjust healthcare, indirect, and total costs (i.e., dependent variables) to covariates, including age, sex, and the Charlson index. Statistical significance was set at a two-sided α = 0.05. All statistical analyses were performed using the SPSSWIN program version 27.

## Results

### Demographic characteristics and comorbidities

**Fig. 2:**
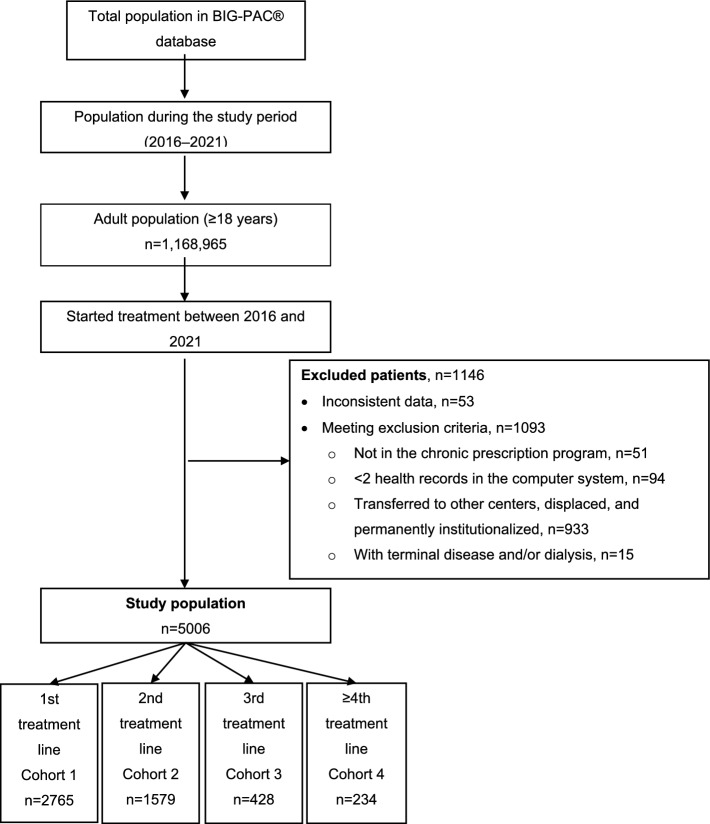
Patient selection flow chart

**Fig. 3:**
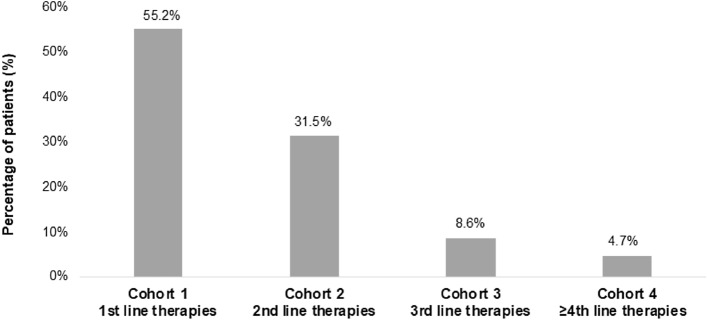
Percentage of patients per group

A total of 5006 patients with epilepsy meeting the inclusion criteria were included and categorized into four groups (i.e., cohorts) according to epilepsy treatment line (Fig. 2). Patients were followed up until death, loss of follow-up or end of the recruitment period (31/12/2021) with a mean (± SD) follow-up time of 3.4 ± 1.6 years in cohort 1, 3.2 ± 1.6 years in cohort 2, 3.2 ± 1.6 years in cohort 3, and 3.1 ± 1.5 years in cohort 4. The characteristics of the study population stratified by treatment line are summarized in Table 1. Most patients were in 1st and 2nd-line treatments (86.7%), and the percentages progressively decreased throughout treatment lines (Fig. 3). Patients were similarly distributed across treatment lines regarding sex and age, with more than half of patients between 18 and 44 years old. The frequencies of comorbidities associated with epilepsy were similar among cohorts, except for depressive syndrome, which was more frequent among patients in the 4th + -line treatment. Hypertension, dyslipidemia, and depressive syndrome were the most frequent comorbidities. Overall morbidity analyses show that the mean number of diagnoses (*p* = 0.037) and the mean Charlson index value (*p* < 0.001) were significantly different across treatment lines, with an increasingly higher Charlson index mean throughout treatment lines. Cohort 4 had a higher percentage of patients with a high Charlson index score (i.e., ≥ 3), with no significant differences between cohorts.

**Table 1:** Demographic characteristics and comorbidities according to treatment line

Study groups	Cohort 1 1st line therapies	Cohort 2 2nd line therapies	Cohort 3 3rd line therapies	Cohort 4 ≥4th line therapies	Total	*P*-value^*^
N, (%)	2765 (55.2)	1579 (31.5)	428 (8.6)	234 (4.7)	5006 (100)	
*Demographic characteristics*						
Age (years), *mean (SD)*	41.3 (15.6)	41.5 (16.1)	40.1 (15.0)	41.1 (13.6)	41.3 (15.6)	0.486
Age (years) categories, n (%)						
18–44 years	1607 (58.1)	924 (58.5)	262 (61.2)	145 (62.0)	2938 (58.7)	0.459
45–64 years	952 (34.4)	513 (32.5)	143 (33.4)	76 (32.5)	1684 (33.6)	
65–74 years	195 (7.1)	129 (8.2)	23 (5.4)	12 (5.1)	359 (7.2)	
≥ 75 years	11 (0.4)	13 (0.8)	0 (0)	1 (0.4)	25 (0.5)	
Sex (female), n (%)	1338 (48.4)	765 (48.4)	209 (48.4)	112 (47.9)	2424 (48.4)	0.996
*General comorbidity*						
Number of diagnoses, *mean (SD)*	1.8 (1.5)	1.8 (1.5)	1.8 (1.8)	2.1 (2.2)	1.8 (1.6)	0.037
Charlson index, *mean (SD)*	0.7 (1.0)	0.8 (1.1)	0.8 (1.1)	1.0 (1.4)	0.8 (1.1)	< 0.001
*Charlson index categories* n (%)						
0	1492 (53.9)	857 (54.2)	247 (57.7)	115 (49.1)	2711 (54.2)	0.114
1	831 (30.1)	451 (28.6)	91 (21.3)	65 (27.8)	1438 (28.7)	
2	220 (8.0)	128 (8.1)	59 (13.8)	19 (8.1)	426 (8.5)	
≥ 3	222 (8.0)	143 (9.1)	31 (6.2)	35 (14.9)	431 (8.7)	
*Epilepsy-associated comorbidities, n (%)*						
Hypertension	884 (31.9)	498 (31.5)	141 (32.9)	89 (38.0)	1612 (32.2)	0.247
Diabetes	427 (15.4)	242 (15.3)	76 (17.8)	48 (20.5)	793 (15.8)	0.130
Dyslipidemia	729 (26.4)	442 (27.9)	113 (26.4)	59 (25.2)	1343 (26.8)	0.628
Obesity	275 (9.9)	145 (9.2)	39 (9.1)	23 (9.8)	482 (9.6)	0.844
Active smoking	290 (10.5)	162 (10.3)	48 (11.2)	24 (10.3)	524 (10.5)	0.952
Alcohol ingestion	54 (1.9)	37 (2.3)	7 (1.6)	5 (2.1)	103 (2.1)	0.769
Ischemic heart disease	162 (5.9)	98 (6.2)	32 (7.5)	21 (8.9)	313 (6.3)	0.188
Stroke	180 (6.5)	115 (7.3)	32 (7.5)	22 (9.4)	349 (6.9)	0.328
Heart failure	99 (3.6)	61 (3.9)	18 (4.2)	13 (5.6)	191 (3.8)	0.469
Kidney failure	118 (4.3)	75 (4.7)	19 (4.4)	11 (4.7)	223 (4.5)	0.900
Asthma	128 (4.6)	82 (5.2)	20 (4.7)	12 (5.1)	242 (4.8)	0.858
Chronic obstructive pulmonary disease	72 (2.6)	35 (2.2)	11 (2.6)	6 (2.6)	124 (2.5)	0.885
Depressive syndrome	625 (22.6)	337 (21.3)	100 (23.4)	72 (30.8)	1134 (22.7)	0.015
Malignant neoplasms	141 (5.1)	96 (6.1)	27 (6.3)	15 (6.4)	279 (5.6)	0.440
Anxiety	545 (19.7)	312 (19.8)	91 (21.3)	53 (22.6)	1001 (19.9)	0.647
Psychoses	42 (1.5)	25 (1.6)	9 (2.1)	6 (2.6)	82 (1.6)	0.470
Attention deficit disorder with hyperactivity	96 (3.5)	45 (2.8)	13 (3.0)	8 (3.4)	162 (3.2)	0.724

^*^ Calculated using ANOVA for continuous variables and the Chi-square test for categorical variables

### Treatment patterns and duration

The distribution of the main antiepileptic drugs prescribed in the study cohorts is summarized in Table 2. In cohort 1, the most frequently used drugs in monotherapy were levetiracetam (28.6%), valproic acid (20.2%), and carbamazepine (10%). In cohort 2, more than 80% of patients received polytherapy, with the most frequently used combinations being levetiracetam + valproic acid (27.5%) or + eslicarbazepine (18.2%) or + carbamazepine (10%). The most frequently used drugs in monotherapy in cohort 2 were levetiracetam (3.9%), valproic acid 3.1%, and lacosamide (2.4%). In cohort 3, less than 10% of patients received monotherapy; the most frequent combinations were levetiracetam + valproic acid + oxcarbazepine (24.3%), levetiracetam + valproic acid + lamotrigine (18.2%), and levetiracetam + valproic acid + perampanel (15.7%). In cohort 4, 100% of the patients received polytherapy; the most frequently used combinations were levetiracetam + valproic acid + oxcarbazepine + zonisamide (37.6%) and levetiracetam + valproic acid + lamotrigine + clonazepam (32.1%).

**Table 2:** Distribution of main antiepileptic drugs prescribed by study cohorts

	N	%
*Cohort 1—1st line therapies, n = 2765*		
Monotherapy		
Levetiracetam	790	28.6
Valproic Acid	558	20.2
Carbamazepine	276	10.0
Lamotrigine	263	9.5
Oxcarbazepine	215	7.8
Eslicarbazepine	196	7.1
Lacosamide	152	5.5
Zonisamide	116	4.2
Phenytoin	62	3.0
Perampanel	55	2.0
Topiramate	41	1.5
Gabapentin	22	0.8
*Cohort 2—2nd line therapies, n = 1579*		
Monotherapy		
Levetiracetam	62	3.9
Valproic Acid	49	3.1
Lacosamide	38	2.4
Lamotrigine	29	1.8
Carbamazepine	28	1.8
Eslicarbazepine	25	1.6
Zonisamide	22	1.4
Polytherapy		
Levetiracetam + Valproic Acid	435	27.5
Levetiracetam + Eslicarbazepine	288	18.2
Levetiracetam + Carbamazepine	158	10.0
Levetiracetam + Lacosamide	127	8.0
Levetiracetam + Perampanel	101	6.4
Valproic Acid + Oxcarbazepine	95	6.0
Valproic Acid + Lamotrigine	83	5.3
Clobazam + combinations	22	1.4
Pregabalin + combinations	5	0.3
*Cohort 3—3rd line therapies, n = 428*		
Monotherapy		
Levetiracetam	11	2.6
Valproic Acid	10	2.3
Polytherapy		
Levetiracetam + Valproic Acid	35	8.2
Levetiracetam + Eslicarbazepine	42	9.8
Levetiracetam + Carbamazepine	32	7.5
Levetiracetam + Valproic Acid + Oxcarbazepine	104	24.3
Levetiracetam + Valproic Acid + Lamotrigine	78	18.2
Levetiracetam + Valproic Acid + Perampanel	67	15.7
Clobazam + combinations	31	7.2
Pregabalin + combinations	3	0.7
*Cohort 4—≥4th line therapies, n = 234*		
Polytherapy		
Levetiracetam + Valproic Acid	12	5.1
Levetiracetam + Carbamazepine	7	3.0
Levetiracetam + Valproic Acid + Oxcarbazepine	15	6.4
Levetiracetam + Valproic Acid + Lamotrigine	10	4.3
Levetiracetam + Valproic Acid + Oxcarbazepine + Zonisamide	88	37.6
Levetiracetam + Valproic Acid + Lamotrigine + Clonazepam	75	32.1
Clobazam + combinations	15	6.4

The duration of epilepsy treatments was calculated from the index date up to the end of follow-up. Treatment duration decreased as treatment lines progressed. The mean (± SD) progression time was 523.2 ± 279.1 days from the 1st to the 2nd treatment line, 351.6 ± 194.4 days from the 2nd to the 3rd line, and 272.7 ± 139.3 days from the 3rd to the 4th + treatment line (Table 3).

**Table 3:** Treatment duration according to treatment line

Cohorts	N, %	Cohort composition	Treatment duration (days), *mean (SD)*
Time from 1st line to 2nd line	2241	Cohort 2, 3, 4 +	523.2 (279.1)
Time from 2nd line to 3rd line	662	Cohort 3, 4 +	351.6 (194.4)
Time from 3rd line to 4th + line	234	Cohort 4 +	272.7 (139.3)

Concomitant medications were analyzed at baseline (during the 6 months prior to index date) and significant differences were found in the administration of psycholeptics between cohorts (*p* = 0.057). Over 65% of the total population used these drugs, reaching a 70.9% use in cohort 4 (Table 4). However, the administration of psychoanaleptics and concomitant medications was similar among cohorts. The most prescribed concomitant medications were drugs for acid-related disorders, anti-inflammatories, antirheumatics, and lipid-modifying agents (Table 4).

**Table 4:** Treatments administered at baseline (previous 6 months from index date), n (%)

Study groups	Cohort 1 1^st^ line therapies	Cohort 2 2^nd^ line therapies	Cohort 3 3^rd^ line therapies	Cohort 4 ≥4th line therapies	Total	*P*-value^*^
N, (%)	2765 (55.2)	1579 (31.5)	428 (8.6)	234 (4.7)	5006 (100)	
Treatments, n (%)						
Psycholeptics (N05)	1814 (65.6)	1006 (63.7)	295 (68.9)	166 (70.9)	3281 (65.5)	0.057
Psychoanaleptics (N06)	1297 (46.9)	736 (46.6)	217 (50.7)	100 (42.7)	2350 (46.9)	0.245
Concomitant medication, n (%)						
Drugs for acid-related disorders (A02)	1234 (44.6)	652 (41.3)	197 (46.0)	101 (43.2)	2184 (43.6)	0.130
Drugs used in diabetes (A10)	375 (13.6)	206 (13)	63 (14.7)	34 (14.5)	678 (13.5)	0.793
Antithrombotic agents (B01)	745 (26.9)	408 (25.8)	112 (26.2)	63 (26.9)	1328 (26.5)	0.879
Antihypertensives (C02)	64 (2.3)	41 (2.6)	12 (2.8)	5 (2.1)	122 (2.4)	0.879
Beta blocking agents (C07)	411 (14.9)	237 (15.0)	73 (17.1)	38 (16.2)	759 (15.2)	0.654
Agents acting on the renin-angiotensin system (C09)	802 (29)	476 (30.1)	127 (29.7)	77 (32.9)	1482 (29.6)	0.589
Lipid modifying agents (C10)	839 (30.3)	498 (31.5)	144 (33.6)	77 (32.9)	1558 (31.1)	0.470
Antiinflammatory and antirheumatic products (M01)	1193 (43.1)	697 (44.1)	204 (47.7)	101 (43.2)	2195 (43.8)	0.364
Number of concomitant medications, mean (SD)	2 (1.9)	2 (2)	2.2 (2.1)	2.1 (2.3)	2.1 (2)	0.565

^*^P-value calculated using Chi-square

### Use of healthcare resources and associated costs

Significant resource use differences among cohorts were found in all the variables analyzed except for computed tomography (Table 5). An increase in hospital admissions and patients on sick leave was observed across treatment lines.

**Table 5:** Resource use per year according to study cohort

Study groups	Cohort 1 1^st^ line therapies	Cohort 2 2^nd^ line therapies	Cohort 3 3^rd^ line therapies	Cohort 4 ≥4th line therapies	TOTAL	*P*-value^*^
N, (%)	2765 (55.2)	1579 (31.5)	428 (8.6%)	234 (4.7%)	5006 (100%)	
Visits and hospitalizations, mean (SD)						
Primary care	10.2 (2.1)	11.3 (2.5)	12.1 (2.5)	13 (2.8)	10.8 (2.4)	< 0.001
Specialist	3.9 (12.4)	5.1 (14.6)	6.0 (13.2)	7.7 (13.2)	4.6 (13.3)	< 0.001
Emergency rooms	1.3 (5.4)	1.0 (5.1)	1.3 (8.8)	1.9 (2.9)	1.3 (5.6)	< 0.001
Hospitalized patients**	278 (10.1)	186 (11.8)	83 (19.4)	51 (21.8)	598 (11.9)	< 0.001
Duration of hospital admissions (days)	0.9 (5.1)	1.2 (6.4)	1.9 (6.0)	2.2 (5.2)	1.1 (5.6)	< 0.001
Laboratory and diagnostic tests, mean (SD)						
Laboratory requests	2.3 (1)	2.5 (1.2)	2.6 (1.1)	2.9 (1.2)	2.4 (1.1)	< 0.001
Radiology	0.6 (0.5)	0.6 (0.5)	0.7 (0.6)	0.7 (0.6)	0.6 (0.5)	< 0.001
Brain computed tomography	0.01 (0.1)	0.01 (0.1)	0.01 (0.1)	0.01 (0.1)	0.01 (0.1)	0.284
Brain magnetic resonance imaging	0.3 (0.6)	0.3 (0.6)	0.3 (0.6)	0.5 (0.6)	0.3 (0.6)	< 0.001
Other	1.7 (1.3)	1.8 (1.3)	2.0 (1.3)	2.2 (1.4)	1.8 (1.3)	< 0.001
Number of concomitant medications, mean (SD)	2.1 (1.5)	2 (1.4)	2.3 (1.6)	2.3 (1.6)	2.1 (1.5)	0.003
Patients with sick leave**	527 (19.1)	327 (20.7)	97 (22.7)	60 (25.6)	1011 (20.2)	0.039
Duration of sick leave (days), mean (SD)	5.5 (23.1)	6.0 (21.5)	6.9 (23.8)	8.2 (22.5)	5.9 (22.6)	0.014

*SD* standard deviation

^*^Calculated using ANOVA for continuous variables and Chi-square for categorical variables

^**^Values expressed as events per 100 patient years (100PY)

**Fig. 4:**
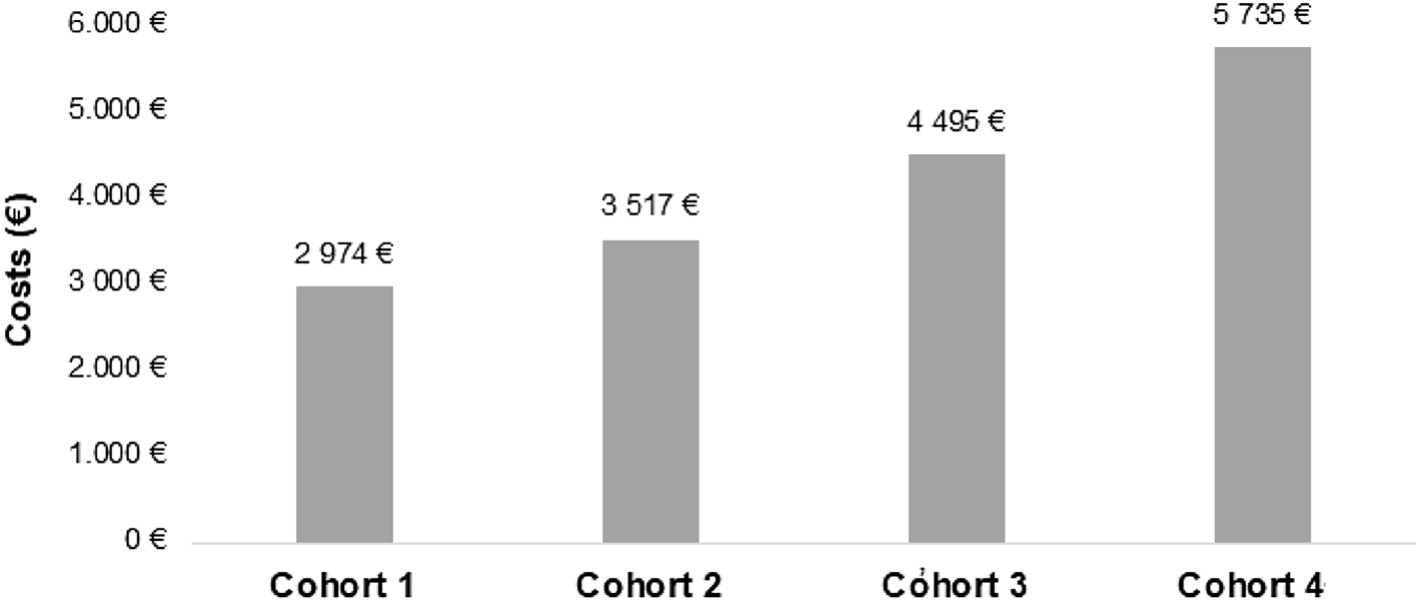
Adjusted total cost according to treatment line

Consistent with the use of resources pattern, the associated costs per patient and year were significantly different among cohorts, showing a progressive increase throughout treatment lines. Treatment cohort 1 showed the lowest costs, with mean (± SD) direct costs of €2414/year (± 4394) and indirect costs of €556.1/year (± 2333), generating a total estimated cost of €2970/per patient and year (± 5111). Treatment cohort 4 showed the highest costs, with direct costs of €4903/year (± 4967), indirect costs of €831.1/year (± 2277), and a total cost of €5734/per patient and year (± 5837) (Table 6). Evaluation of costs adjusted by age, sex, and Charlson index confirmed the increase in direct and total costs throughout treatment lines, with a mean difference of €2761 in total costs between cohorts (*p* < 0.001). The mean adjusted total costs were €2974 (95% CI 2773–3175) in cohort 1, and €5735 (95% CI 5043–6428) in cohort 4 (Fig. 4). However, no statistically significant differences in indirect costs were found among cohorts. The highest direct costs were epilepsy medication (overall mean cost of €959.6/year [± 2704]), followed by days at the hospital (overall mean cost of €552.9/year [± 2710]), and specialist visits (overall mean cost of €425.1/year [± 1220]).

**Table 6:** Healthcare and indirect costs (euros) according to study cohorts per patient and year, mean (SD)

Study groups	Cohort 1 1st line therapies	Cohort 2 2nd line therapies	Cohort 3 3rd line therapies	Cohort 4 ≥4th line therapies	Total	*P*-value*
N, (%)	2765 (55.2)	1579 (31.5)	428 (8.6)	234 (4.7)	5006 (100)	
Primary care visits	236.8 (48.6)	262.5 (58.5)	279.6 (58.9)	302.3 (64.1)	251.7 (56.7)	< 0.001
Specialized visits	354.4 (1138)	470.9 (1344)	556.4 (1219)	710.1 (1215)	425.1 (1220)	< 0.001
Emergency rooms visits	157.6 (638)	120.2 (603.8)	156.3 (1035.9)	220.2 (348.9)	148.6 (661.4)	< 0.001
Laboratory requests	51.2 (23.1)	54.9 (25.8)	57.8 (25.1)	66.3 (25.9)	53.6 (24.6)	< 0.001
Radiology requests	10.7 (9.3)	11.3 (9.9)	13.3 (10.9)	12.9 (11.5)	11.2 (9.8)	< 0.001
Brain computed tomography	0.8 (6.5)	0.7 (6.3)	0.9 (5.8)	1.0 (6.2)	0.8 (6.4)	0.284
Brain magnetic resonance imaging	47.9 (115.9)	47.9 (99.5)	48.8 (105.4)	84.2 (109.2)	49.7 (110)	< 0.001
Other tests	64.1 (47.4)	68.2 (49.9)	73.3 (50.1)	82.3 (51.9)	67.0 (48.5)	< 0.001
Days at hospital	438.1 (2437)	577.5 (3092)	934.2 (2907)	1046 (2525)	552.9 (2710)	< 0.001
Epilepsy medication	777.7 (2488)	1008 (3057)	1374 (2313)	2023 (2964)	959.6 (2704)	< 0.001
Concomitant medication	274.2 (480.2)	274.9 (424.6)	334.9 (459.2)	354.8 (462.1)	283.4 (461.2)	< 0.001
Healthcare costs	2414 (4394)	2897 (5230)	3829 (4715)	4903 (4967)	2803 (4766)	< 0.001
Indirect costs	556.1 (2333)	610.5 (2179)	701.4 (2409)	831.1 (2277)	598.5 (2290)	0.014
Total costs	2970 (5111)	3508 (5798)	4530 (5586)	5734 (5837)	3402 (5454)	< 0.001
Adjusted costs, (mean 95% CI)**					Mean differences***	*P*-value**
Healthcare costs	2416 (2239–2592)	2899 (2666–3132)	3823 (3375–4271)	4894 (4287–5501)	2478	< 0.001
Indirect costs	558.6 (474.7–642.6)	618.3 (507.3–729.4)	672.2 (458.9–885.6)	841.7 (552.7–1131)	283.1	0.245
Total costs	2974 (2773–3175)	3517 (3251–3783)	4495 (3984–5006)	5736 (5043–6428)	2761	< 0.001

Propensity score matching

*ANCOVA* analysis of the covariance, *CI* confidence interval, *SD* standard deviation

^*^Calculated using analysis of the variance (ANOVA)

^**^Calculated using ANCOVA, including age, sex, and Charlson index as covariates

^***^Cohort 1 vs cohort 4

## Discussion

This study provides real-world evidence on the characteristics, comorbidities and most frequently used treatments of patients with epilepsy in Spain. We also evaluated the duration of the therapy lines, and the progression of healthcare resource use and associated costs across epilepsy treatment lines.

Study cohorts were defined according to treatment line and our results show that the patients’ characteristics were comparable among them. Comorbidities were similar among cohorts when individually compared, except that depressive syndrome was more prevalent in cohort 4. However, when the Charlson Index, a weighted index to predict risk of death for patients with specific comorbid conditions was calculated, a trend towards a higher comorbidity burden was observed with treatment line progression, with a significantly higher score in cohort 4. This agrees with the previously described higher comorbidity burden in patients with medically refractory epilepsy [30].

Previous studies reported that approximately a third of patients remain uncontrolled after treatment with at least two antiepileptic drugs [12–14]. In our study, the proportion of patients in the ≥ 3rd line of treatment was 13.3%. However, in cohorts 1 and 2 of our study, a high proportion of patients had a follow up that was shorter than the average time to progress to the next line of treatment. Therefore, DRE could not be ruled out in these patients.

Patients with newly diagnosed focal epilepsy have been shown to have a higher prevalence of mood disorders, anxiety disorders and suicidality than the general population [31]. In addition, studies have shown an association between DRE and depressive disorders [32, 33]. In agreement, our results showed that 30.8% of the patients in the 4th + treatment line, in which 93.2% of the patients were prescribed ≥ 3 antiepileptic drugs, were diagnosed with depressive disorder as compared with 22.6%, 21.3% and 23.4% in the 1st, 2nd and 3rd treatment lines, respectively. For patients with DRE, depression is a significant predictor of reduced quality of life [34]. Therefore, special attention must be given to prevent depressive disorder during the management of patients with epilepsy, not only in those with DRE but also in patients with newly diagnosed disease.

Analysis of the main ASMs prescribed during the study period revealed a broad heterogeneity in drug combinations used from the second line onwards, in line with a lack of a specific therapeutic algorithm. Many patients were treated with levetiracetam, in accordance with a previous study [35]. We also observed widespread use of the first-generation drug valproic acid. This might be due to its high effectiveness against generalized seizure and related epileptic syndromes [36], despite the restrictions in women with childbearing potential [37]. For third-generation drugs such as brivaracetam, which have only become available in Spain more recently, prescriptions recorded in our study may be lower than current usage based on the time period of the study. We also observed scarce use of valproate + lamotrigine in the 3rd and ≥4th lines of therapy compared with other polytherapies, despite this combination being shown to be effective in the management of patients with refractory epilepsy based on the synergistic effect of the two agents [38].

Our data show a decrease in treatment duration as treatment lines progressed. This could be due to the perception of poor disease control by the physician, whose alternative might be moving the patient to the following treatment line. This reduction in treatment line duration was accompanied by an increase in HRU use and associated costs. Our results corroborate that poor control of epilepsy involves substantial costs; the incremental cost between lines 1 and 2 was smaller than between lines 2 and 3, which in turn was smaller than between lines 3 and 4, thus indicating that costs progressively increased across treatment lines. Moreover, the cost difference widened with each line (the difference between lines 3 and 4 was bigger than between lines 1 and 2). In this regard, other studies found that DRE was associated with increased expenditure. Villanueva et al. described that the patients diagnosed with DRE had higher direct epilepsy-related costs than non-DRE patients [18]. Similarly, Willems et al. found that, compared with non-DRE, DRE entailed higher expenditure in terms of total, direct, and indirect costs of illness [39]. Furthermore, Zelicourt et al. reported that the use of almost all healthcare resources was higher in patients with DRE; consequently, the direct epilepsy-related costs were more than double that in non-DRE patients [40]. Similarly, DRE costs in Spanish daily clinical practice have been shown to be associated with lower patient-reported outcome scores, highlighting how the increasing negative impact of DRE on the patient leads to higher costs [41].

The mean annual direct cost per patient of €2803 found in our study is consistent with that reported by other authors (range €1698–5432 per patient and year) [20, 39, 42, 43]. In addition, the estimated mean annual direct cost per patient was €3829 and €4903 for patients receiving 3rd and ≥4th lines of treatment, respectively, which are in line with those found in other studies (range €3777–6304 per patient and year) [18, 40, 44]. Direct medical costs increased with the treatment line and the main component of these direct costs was pharmacological treatment. This agrees with previously published data showing that the number of antiepileptic drugs, seizure frequency, and disease duration are significantly associated with the cost of illness of epilepsy [45]. In contrast with the recommendations of epilepsy guidelines [5, 9, 10], our data shows that 533 out of 662 patients in 3rd and ≥4th line therapy (85.7%) were treated with 3–4 antiepileptic medications, increasing the direct costs associated with the management of the disease. In addition, the number and duration of hospitalizations also increased with treatment line, which also explains the increase in economic burden in subsequent treatment lines.

The potential confounding effect of age, sex, and comorbidities on direct and indirect costs was addressed with an adjusted analysis. The difference in total and direct costs among cohorts remained statistically significant after adjustment, but the difference in indirect costs did not. In this regard, our analysis of indirect costs did not include unemployment or early retirement, which have been described as major contributors to indirect costs [39, 43, 46, 47]. Furthermore, indirect costs among cohorts were adjusted for comorbidities, including depression, even though depressive disorders are known to be associated with DRE [32, 33] and with an increased risk of sick leave [48], which in turn has an impact on epilepsy-associated indirect costs. In our study, the prevalence of depressive syndrome increased significantly in patients with more complex disease and associated advanced lines of treatment. Even after adjusting for depression, there was a noticeable trend toward increased indirect costs with later lines of therapy. Together, our direct and indirect cost data indicate that delaying epilepsy control increases the burden of epilepsy for the healthcare system and society. While little progress has been made to improve seizure-free rates among patients in the last few decades, use of new ASMs at earlier lines of therapy may provide an opportunity to improve seizure control [49], thereby reducing costs. Notably, new ASMs such as cenomabate (for focal seizures) and fenfluramine (for developmental and epileptic encephalopathy) were not commercialized in Spain at the time of database closure (December 2021) and are not therefore factored into our analysis.

This study has some limitations. First, the BIG-PAC^®^ database is administrative and may lack some data about the study population, especially if patients have been treated in private healthcare centers or in public healthcare centers that are out of the scope of BIG-PAC^®^. The missing data may lead to classification bias and errors in categorizing diseases and the operational extent of costs. Another limitation is that the classification of patients by pathology in the BIG-PAC^®^ database is based on the ICD-9-CM coding system and not on the ILAE classification. Moreover, our study did not include direct non-healthcare costs (i.e., out-of-pocket costs or those paid for by the patient/family) as they are not registered in the database and the study design does not provide direct access to patients. Another significant limitation is the lack of information about the reasons for treatment change, which has prevented us from discriminating treatment failures from other reasons for treatment change and, therefore, calculating the proportion of DRE patients. Moreover, we have no information on how many patients are treated in epilepsy referral centers, which might impact on clinical practice. However, there are several strengths as this study included a large sample of patients with epilepsy, allowing us to capture valuable data on the public Spanish National Health System and to analyze the use of resources and costs by treatment line. In addition, we assessed not only direct, healthcare-related costs but also indirect costs associated with productivity loss. Although the differences between healthcare systems may hinder the application of our results to other settings, this analysis highlights the importance of early control of epilepsy to reduce the use of resources and the costs associated with this disease.

## Conclusions

In conclusion, this study confirmed the substantial expenditure and use of resources derived from managing patients with epilepsy and found a progressive increase in the use of resources and costs across subsequent treatment lines. Consequently, the early control of epilepsy may not only benefit patients but also reduce the economic burden for healthcare providers.

### Supplementary Information

Below is the link to the electronic supplementary material.

Supplementary file1 (DOCX 36 KB)

## Data Availability

All of the data used for this study is available from BIG-PAC^®^, a dissociated and anonymized administrative database; the secondary data used in this study are not linked to patients’ identity and may be shared at the request of any qualified investigator for purposes of replicating procedures and results.
