# Supplementary material for: Clinical and economic implications of epilepsy management across treatment lines in Spain: a real-life database analysis
Source: J Neurol. 2023 Aug 25;270(12):5945–57. doi: 10.1007/s00415-023-11958-x (PMC10632298; doi:10.1007/s00415-023-11958-x)
Supplement: Supplementary file 1 — Supplementary file1 (DOCX 36 KB) [file 415_2023_11958_MOESM1_ESM.docx]

# Supplementary Tables

**Table S1.** Comorbidities codification according to the ICD-9-CM coding system

| **ICD-9-CM codes** | **Comorbidity** |
| --- | --- |
| 410 | Hypertension |
| 250 | Diabetes |
| 272 | Dyslipidemia |
| 278 | Obesity |
| 305.1 | Active smoking |
| 303.9 | Alcohol ingestion |
| 410 – 414 | Ischemic heart disease |
| 434.91 | Stroke |
| 428 | Heart failure |
| 593.9 | Kidney failure |
| 493 | Asthma |
| 496 | Chronic Obstructive Pulmonary Disease |
| 311 | Depressive syndrome |
| 140 – 239 | Malignant neoplasms |
| 300.0 | Anxiety |
| 298 | Psychoses |
| 314.01 | Attention deficit disorder with hyperactivity |

| **Table S2.** Charlson Comorbidity Index | | |
| --- | --- | --- |
| **ICD-9-CM codes** | **Condition** | **Score** |
| 410 – 410.9, 412 | Myocardial infarction | 1 |
| 428 – 428.9 | Congestive heart failure | 1 |
| 433.9, 441 – 441.9, 785.4, V43.4 | Peripheral vascular disease | 1 |
| 430 – 438 | Cerebrovascular disease | 1 |
| 290 – 290.9 | Dementia | 1 |
| 490 – 496, 500 – 505, 506.4 | Chronic pulmonary disease | 1 |
| 710.0, 710.1, 710.4, 714.0 – 714.2, 714.81, 725 | Rheumatological disease | 1 |
| 531 – 534.9 | Peptic ulcer | 1 |
| 571.2, 571.5, 571.6, 571.4 – 571.49 | Mild liver disease | 1 |
| 250 – 250.3, 250.7 | Diabetes | 1 |
| 250.4 – 250.6 | Diabetes with chronic complications | 2 |
| 344.1, 342 – 342.9 | Hemiplegia or paraplegia | 2 |
| 582 – 582.9, 583 – 583.7, 585, 586, 588 – 588.9 | Kidney disease | 2 |
| 572.2 – 572.8 | Moderate or severe liver disease | 3 |
| 042 – 044.9 | AIDS | 6 |

**Table S3.** ICD-9-CM codes for epilepsy surgeries

| **ICD-9-CM codes** | **Procedures** |
| --- | --- |
| 01.52 | Hemispherectomy |
| 01.53 | Lobectomy of brain |
| 17.61 | Laser interstitial thermal therapy |
| 92.21 | Superficial radiation |
| 92.29 | Other radiotherapeutic procedure |
| 92.31 | Single-source photon radiosurgery |
| 92.32 | Multi-source photon radiosurgery |
| 92.33 | Particulate radiosurgery |
| 92.39 | Stereotactic radiosurgery, not elsewhere classified |

**Table S4.** Unitary costs and productivity based on Spanish National Health System (Year 2020)

| **Sanitary and not-sanitary resources** | **Unit costs (€)** |
| --- | --- |
| **Medical visits** |  |
| Primary care | 23.2 |
| Emergency visits | 117.5 |
| Hospitalization | 480.9 |
| Specialist visit^*^ | 92.0 |
| **Complementary visits** |  |
| Laboratory tests | 22.3 |
| Conventional radiology | 18.5 |
| Brain computed tomography | 96.0 |
| Brain magnetic resonance imaging | 177.0 |
| Electroencephalography | 37.1 |
| Pharmaceutic prescription | RRP + taxes |
| **Laboral productivity - indirect costs^**^** |  |
| Cost per day not worked | 101.2 |

RRP: recommended retail price.

^*^ Considered: Psychiatry, Psychology, and Neurology departments.

^**^ Estimation of the average earnings by age and sex in Spanish active population.
